# Association between Dental Amalgam Filling and Essential Tremor: A Nationwide Population-Based Case Control Study in Taiwan

**DOI:** 10.3390/ijerph17030780

**Published:** 2020-01-27

**Authors:** Chien-Fang Tseng, Kun-Huang Chen, Hui-Chieh Yu, Yu-Chao Chang

**Affiliations:** 1 School of Dentistry, Chung Shan Medical University, Taichung 40201, Taiwan; chenfang8858@gmail.com (C.-F.T.); yujessica7@gmail.com (H.-C.Y.); 2 Department of Artificial Intelligence, CTBC Business School, Tainan 709, Taiwan; khchen@ctbc.edu.tw; 3 Department of Dentistry, Chung Shan Medical University Hospital, Taichung 40201, Taiwan

**Keywords:** essential tremor, dental amalgam fillings, nationwide population, case-control study

## Abstract

Essential tremor (ET) is a common neurological disorder and the most common movement disorder. Low-level occupational exposure to mercury vapor is known to be a crucial factor that increases the risk of tremor. Dental amalgam is one of the main sources of mercury in those who possess amalgam restorations. However, the relationship between ET and amalgam filling (AMF) is not quite clear. The purpose of this study was to investigate the association between AMF and the risk of ET using a population-based administrative databank. The data for this study were sourced from the Taiwanese National Health Insurance Research Database (NHIRD). A retrospective case-control study was conducted using this databank from 2000 to 2013. Case and control groups were matched by sex, age, urbanization level, monthly income, and Charlson comorbidity index using the propensity score method with a 1:1 ratio. In this study, 3008 cases and 3008 controls were included. The results from this nationwide population-based case-control study did not indicate any association between ET and AMF in Taiwan. Although the results were not significantly statistical, the findings may be worthy to be valued.

## 1. Introduction

Tremor is defined as an involuntary, rhythmic, and oscillatory movement of a body part [1]. The most prevalent type of tremor is a bilateral action tremor of the arms, which is often diagnosed as an essential tremor (ET). The following are the criteria for ET: isolated tremor syndrome characterized by bilateral upper limb action tremor; duration of at least three years; with or without tremor in other locations (e.g., head, voice, or lower limbs); and absence of other neurological signs, such as dystonia, ataxia, or Parkinsonism [2]. ET affects about 1% of those aged ≥60 years old [3]. Genetic and environmental risk factors may affect cerebellar and olive function, leading to cortical–cerebellar–thalamic activity abnormalities and ultimately resulting in ET [4].

Dental amalgam contains about 50% mercury and has been a common dental restorative material. Traditional amalgam, which contains an amount of copper lower than 6 mass%, is classified as low-copper amalgam. Starting in the 1960s, high-copper dental amalgams were developed, which contain greater than 8–10 mass% copper [5]. However, in acidic conditions, high-copper-formulation amalgams have demonstrated higher mercury dissolution [6]. Recently, increased instability and higher emission of mercury vapor were reported in high-copper amalgams [7]. Judging from the age range involved, many would have been filled with high-copper amalgam from the late 1960s. This is known to corrode at a greater rate and for longer than the earlier composition type.

Human mercury body burden due to dental amalgam filling (AMF) has been proved by autopsy studies [8]. Higher amounts of mercury deposited in the brain and kidney tissues were correlated with AMF [9,10,11,12]. Teeth restored with amalgam have positive correlations with the mercury level found in patients’ blood and urine [13,14,15]. In addition, a recent prevalence study, for which 2137 subjects were recruited, revealed that an individual with seven or more dental amalgam surfaces could have 30–50% higher urinary mercury levels than an individual without AMF [16].

The exact mechanisms contributing to the pathogenesis of ET still remain unclear. Previous studies have shown a positive association between occupational exposure to mercury and tremor for those employed as chloralkali workers, fluorescent lamp production factory workers, and dentists [17,18,19,20]. Although the results from the two studies demonstrated no significant differences in profiles of tremor assessments in workers exposed to mercury vapor [21,22], no study has been made to show the link between ET risk and AMF. The modern high-copper amalgams now available on the market have been reported to have significant mercury emissions [7]. The purpose of this study was to evaluate the relationship between AMF and ET in Taiwan. Therefore, we conducted a population-based case-control study to investigate this putative association using the National Health Insurance Research Database (NHIRD).

## 2. Materials and Methods

### 2.1. Data Source and Study Design

The dataset used in this case-control study was the Longitudinal Health Insurance Database 2010 (LHID2010), created and released to the public by the National Health Research Institute (NHRI) in Taiwan. LHID2010 includes all the original claims data and registration files from 2000 to 2013 for 1 million individuals randomly sampled from the Registry for Beneficiaries of the National Health Institute (NHI) program in 2010. This compulsory NHI program, with a coverage rate of up to 99.9% of the Taiwanese population, was instituted in 2014 [23]. The disease diagnoses were defined according to the International Classification of Diseases, Ninth Revision, Clinical Modification (ICD-9-CM). This study was approved by the Chung Shan Medical University Hospital Ethics Review Board (CSMUH No. CS2-17086). Because the LHID2010 consists of de-identified secondary data released to the public for research purposes, written informed consent was not required.

### 2.2. Selection of Case and Control Groups

**Figure 1:**
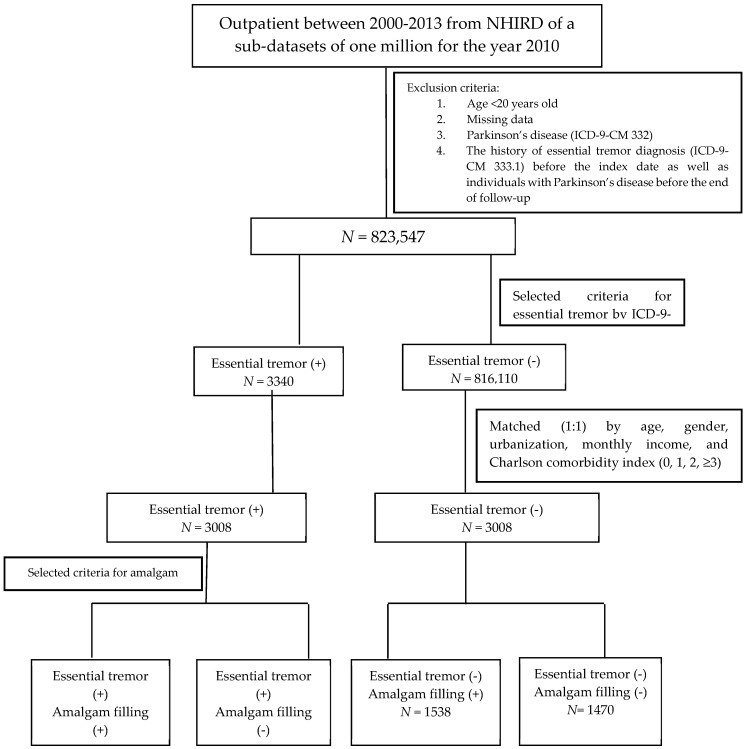
The flowchart of the study.

Data on the individuals used for this case-control study were retrieved from the LHID2010 for the period between January 2000 and December 2013. In order to limit our study sample to the adult population, the subjects who were aged less than 20 years old were excluded. Individuals withdrew from the NHI program, or had missing data in LHID2010 were not included in this study. Patients who had a past history of Parkinson’s disease (ICD-9-CM 332) were also excluded to ensure the accuracy of the ET diagnosis. In addition, patients diagnosed with ET (ICD-9-CM 333.1) before a history of AMF were also excluded. Moreover, we only included those subjects who had at least three consensus diagnoses of ET in order to increase the validity of the ET diagnoses sourced from the LHID2010. For each case, we conditionally selected comparison subjects from the general population matched by sex, age, urbanization level, socioeconomic status, and Charlson comorbidity index (CCI) using the propensity score method at a 1:1 ratio. The Charlson score was categorized as 0, 1, 2, and ≥3, with higher scores indicating greater comorbidity [24]. The flowchart of the study is shown in Figure 1.

### 2.3. Exposure Assessment

To validate the AMF sourced from LHID2010, we identified AMF cases by treatment codes (89001C, 89002C, 89003C, 89101C, 89102C, and 89103C) of the NHI system (Table 1). Potential confounding factors, including sex, age, socioeconomic factors, urbanization, and CCI, were identified and categorized, as shown in Figure 1.

### 2.4. Statistical Analysis

Data analyses were performed using SPSS version 18 (SPSS, Chicago, IL, USA). Statistical analyses were performed using Student’s *t*-test for continuous variables and the chi-squared test for categorical variables. The odds ratio (OR) between case and control groups was analyzed by the chi-squared test. A multivariate logistic regression model was performed for subgroup analysis. All results are presented as ORs and 95% confidence intervals (CIs). Adjustments were made for age, sex, income, region, and CCI. Statistical significance was set at *p* < 0.05.

## 3. Results

Totals of 3008 cases and 3008 control patients were included in this study. In Table 2, the data show that the individuals had an average age of 65.40 ± 17.54 years. In addition, 3373 (56.07%) cases were female and 2643 (43.93%) were male. Demographic characteristics, including age, sex, urbanization, region, monthly income, and CCI score, were not significantly different between the cases and control groups (*p* > 0.05).

As shown in Table 3, the research conducted did not reveal any direct relationship between the risk of ET and those with AMF (adjusted OR: 0.94, 95% CI = 0.85–1.04). 

The risk of ET with AMF stratified by sex is shown in Table 4. ET was not associated with AMF regardless of sex. The adjusted OR of AMF for ET was 0.92 (95% CI = 0.80–1.05) and 0.97 (95% CI = 0.83–1.13) than non-AMF for women and men, respectively.

## 4. Discussion

To the best of our knowledge, this study offers the first considerable understanding of the association between AMF and ET using a large-scale population-based dataset. In this case-control study, we found that AMF was not positively related to the risk of ET. This case-control study had more strength than previous cross-sectional designs regarding the association between AMF and ET. With the nationwide population derived from the NHIRD, this longitudinal sampling dataset from 2000 to 2013 made the study more representative. 

In this study, to ensure the accurate diagnosis of ET, patients who had a past history of Parkinson’s disease were excluded. Diagnosed essential tremor patients before a history of amalgam filling were also excluded. Only patients with at least three consensus diagnoses were captured. The validity of the AMF was also confirmed by the routine verification of treatment codes of the NHI system for health insurance reimbursement eligibility. Salient and meaningful findings were exhibited in this nationwide registry-based study.

All types of dental amalgams contain mercury, which is partly emitted as mercury vapor. The toxicity of mercury could lead to cell damage by increasing oxidative stress [25]. Magnetic resonance spectroscopy has shown decreased levels of N-acetylaspartate (NAA) in the cerebellum, a finding that indicates a loss or dysfunction of neurons [26]. Increased γ-aminobutyric acid (GABA) dysfunction [27] has also been revealed in the cerebellum of persons with essential tremor. Occupational mercury exposure was associated with the occurrence of tremor [17,18,19,20], but not in some studies [21,22]. However, there is not a positive relation between ET and AMF in the present study. The levels of mercury exposure may be different between occupational mercury exposure and AMF. Mercury might be leached during chewing and brushing from the surface of AMF bearers. However, it is still without any exposure assessment of mercury vapor from amalgam restorations in vivo. 

Some potential limitations should be noted regarding the use of claims databases. First, family history, symptoms, and ET severity are not available in the NHIRD. In addition, information about daily diet, the brands of amalgam, and the amalgam formulation were not clear. This may result in compromised findings and inadequate adjustment for confounding factors. Second, the location of the AMF could not be truly interpreted by the treatment codes of the NHI system. Third, other teeth metal restorations, such as inlays or crowns, were also not obtained from the NHIRD. Fourth, the weakness of propensity matching methodology nature which does not account for unmeasured confounders in this study should be taken into consideration.

## 5. Conclusions

Taken together, this nationwide population-based case-control study revealed no association between ET and AMF in Taiwan. Dental amalgams could be a potential source of mercury exposure in the current environment. It is essential to assess the potential neurotoxicity from amalgam-related mercury. A further risk-benefit analysis would require consideration of efficacy, longevity and performance of dental amalgam.

## Figures and Tables

**Table 1:** National health insurance treatment codes for an amalgam filling.

Code	Indication
89001C	Single-surface amalgam restoration
89002C	Two-surface amalgam restoration
89003C	Three-surface amalgam restoration
89101C	Single-surface amalgam restoration in specific cases
89102C	Two-surface amalgam restoration in specific cases
89103C	Three-surface amalgam restoration in specific cases

Specific cases refer to special needs, chemotherapy, and radiotherapy.

**Table 2:** Demographic characteristics of individuals in this study.

	Total (N = 6016)		Essential Tremor (*n* = 3008)		Non- Essential Tremor (*n* = 3008)		*p*-value
	Population	%	Population	%	Population	%	
**Amalgam filling**	247	4.11%	132	4.39%	115	3.82%	
**Age**	65.4 ± 17.5		65.2 ± 17.7		65.6 ± 17.4		
**Age groups**							0.474
20–29	247	4.11%	132	4.39%	115	3.82%	
30–39	432	7.18%	223	7.41%	209	6.95%	
40–49	485	8.06%	234	7.78%	251	8.34%	
50–59	811	13.48%	410	13.63%	401	13.33%	
60–69	1085	18.04%	513	17.05%	572	19.02%	
70–79	1497	24.88%	771	25.63%	726	24.14%	
≥80	1459	24.25%	725	24.10%	734	24.40%	
**Sex**							0.665
Female	3373	56.07%	1671	55.55%	1702	56.58%	
Male	2643	43.93%	1337	44.45%	1306	43.42%	
**Urbanization**							0.724
Urban	3801	63.18%	1902	63.23%	1899	63.13%	
Suburban	1668	27.73%	840	27.93%	828	27.53%	
Rural	547	9.09%	266	8.84%	281	9.34%	
CCI							0.052
0	798	13.26%	325	10.80%	473	15.72%	
1	1023	17.00%	519	17.25%	504	16.76%	
2	1003	16.67%	526	17.49%	477	15.86%	
≥3	3192	53.06%	1638	54.45%	1554	51.66%	
**Monthly income**							0.849
<NT$ 20,000	4691	77.98%	2349	78.09%	2342	77.86%	
NT$ 20,000~40,000	839	13.95%	417	13.86%	422	14.03%	
>NT$ 40,000	486	8.08%	242	8.05%	244	8.11%	

Abbreviations: CCI—Charlson comorbidity index. Statistical analysis was performed by using Student’s t-test.

**Table 3:** Odds ratio for amalgam filling of those with a diagnosis of essential tremor.

	With ET (*n* = 3008)		Without ET (*n* = 3008)	
	No. of Patients	%	No. of Patients	%
Non-AMF	1507	50.10%	1470	48.87%
AMF	1501	49.90%	1538	51.13%
OR (95% CI)	0.95 (0.86–1.05)		1.00	
Adjusted OR (95% CI)	0.94 (0.85–1.04)		1.00	

Abbreviations: ET—essential tremor; AMF—amalgam filling; OR—odds ratio; CI—confidence interval. Adjustment by age, gender, urbanization, CCI, and monthly income.

**Table 4:** Odds ratio for sex of those with a diagnosis of essential tremor with an amalgam filling.

	With ET	Without ET	OR (95% CI)	Adjusted OR (95% CI)
Sex	No. of AMF	No. of AMF		
**Female**	851	802	0.93 (0.81–1.06)	0.92 (0.80–1.05)
**Male**	650	638	0.99 (0.85–1.15)	0.97 (0.83–1.13)

Abbreviations: ET—essential tremor; AMF—amalgam filling; OR—odds ratio; CI—confidence interval. Adjustment by age, urbanization, CCI, and monthly income.
